# What is being measured by formal thought disorder scales? An item-level content analysis

**DOI:** 10.1017/S0033291726104152

**Published:** 2026-04-13

**Authors:** Vanteemar S. Sreeraj, Alban Voppel, Ganesan Venkatasubramanian, Lena Palaniyappan

**Affiliations:** 1 Department of Psychiatry, National Institute of Mental Health and Neuro Sciences (NIMHANS), Bengaluru, India; 2 Douglas Mental Health University Institute, Department of Psychiatry, McGill University, Montreal, Quebec, Canada; 3 Department of Psychology, McGill University, Montreal, Quebec, Canada; 4 Department of Psychiatry, Western University, London, Ontario, Canada

**Keywords:** checklist, communication disorder, language disorder, psychopathology, psychoses, thought disorder

## Abstract

**Background:**

Formal thought disorder (FTD), characterized by disruptions in the flow and form of thought, is a core feature of psychosis. But its measurement is fragmented across numerous rating scales, leading to its continued neglect in both research and clinical practice. To determine if different FTD scales measure the same underlying construct, we need to assess the degree to which the content of commonly used FTD scales overlaps with each other.

**Methods:**

We conducted a systematic review to identify all standardized, clinician-rated scales used to measure FTD in psychotic disorders. From this set, we extracted individual items and derived a consensus list of 56 discrete FTD phenomena. Two independent clinical experts conducted item-to-item mapping for every scale item onto these FTD phenomena. Content overlap between scales was quantified using Jaccard Similarity Index (JSI). We determined the overall coverage achieved via several combinations of FTD scales.

**Results:**

The 15 scales, comprising 207 items, showed weak content overlap. The mean JSI across all scale pairs was low, and no single phenomenon featured across all scales. While some core FTD phenomena (e.g. ‘incoherence’, ‘poor speech content’, ‘drifting-off’) were represented in many scales, 20% of all identified features were idiosyncratic, appearing in only one scale.

**Conclusions:**

Existing FTD rating scales capture a wide but heterogeneous array of symptoms with poor content overlap. This lack of harmonization challenges the comparability of mechanistic and interventional studies. We highlight the need for a consensus-based, standardized measurement of FTD and provide a comprehensive checklist to advance the research and clinical practice.

## Introduction

Formal thought disorder (FTD), a core manifestation of psychosis, is characterised by disorganised speech reflecting breakdown in the form and flow of thought (Kircher, Bröhl, Meier, & Engelen, 2018). It is a key predictor of functional impairment (Marggraf, Lysaker, Salyers, & Minor, 2020; Norman et al., 1999) and presents a significant therapeutic challenge. Despite its clinical importance, the measurement of FTD is a formidable problem as it is a syndrome of variously labelled clinical signs, and existing rating scales operationalize these signs through divergent theoretical frameworks and item pools (Barrera, 2025). This heterogeneity parallels the difficulties seen in measuring other complex constructs like depression (Fried, 2017) and clinical high-risk states (Bernardin, Gauld, Martin, Laprévote, & Dondé, 2023) where lack of convergence among measurement tools hinders the synthesis of evidence. At present, we do not know whether various FTD scales measure the same underlying phenomena, and to what degree their findings can be pooled in meta-analyses.

The lack of a standardized measurement approach has created a void in the FTD literature, impeding progress in understanding its mechanisms and developing targeted interventions. For example, large-scale synthesis of studies investigating FTD’s neural substrates remains predominantly descriptive (Cavelti, Kircher, Nagels, Strik, & Homan, 2018; Sumner, Bell, & Rossell, 2018a, 2018b), precluding the generation of strong testable theories. The central problem lies in the unknown interchangeability of these scales; without understanding how individual scale items map onto a common set of symptoms (or signs, to be more precise), we cannot determine if they are measuring congruent or divergent aspects of the heterogeneous FTD construct. In this work, we aim to systematically investigate the item-level content overlap across commonly used FTD rating scales.

We seek to determine (1) how many distinct features comprise the broad construct of FTD as currently measured, (2) which of these features are shared across scales; and ultimately, and (3) what degree of interchangeability exists among the existing measurement instruments. By doing so, we generate a comprehensive inventory of FTD items – a key priority for clarifying the measurement of FTD (see our detailed companion review, Palaniyappan, Sreeraj, Venkatasubramanian, & Voppel, 2026). This work clarifies the conceptual transportability of the FTD literature and points to appropriate use for the available instruments. Identifying items with high overlap will help in weighting the items that constitute FTD, thereby guiding the harmonization of measurements in the future. This is a crucial step toward improving the replicability and generalizability of findings related to mechanistic and interventional studies in FTD.

## Methods

### Identifying FTD rating scales

A systematic survey was conducted in PubMed and Psynet (APA) databases to identify the tools used to measure the thought disorder rated by clinicians. Following search terms were used: (‘formal thought disorder’ OR ‘thought disorder’ OR (‘disorg*’ AND (‘thought’ OR ‘speech’ OR ‘concept*’ OR ‘cognitive’))) AND (‘psychosis’ OR ‘schizo*’ OR ‘Bipolar Disorder’ OR ‘Mania’ OR ‘Affective disorder’ OR ‘Depress*’ OR ‘Anxiety’ OR ‘Obsess*’ OR ‘Personality Disorder’) with appropriate filters to include clinical studies with abstracts in English up to December 2023. We included original papers published in a peer-reviewed, indexed journal where the study used an operationalized observer-rated/interview-based psychopathology rating tool to assess formal thought disorder in at least one psychiatric disorder or clinically high-risk group with abstract and/or publication describing the scale available in the English language. Studies that did not involve populations with psychiatric disorders or clinically high-risk populations as well as publications that were case reports, conference presentations, and reviews/meta-analyses, were also excluded.

Two authors, VSS and LP, independently screened all the abstracts and papers for eligibility. Finally, 254 publications were selected based on consensus (PRISMA flow chart in Supplementary Figure S1). The clinical rating scales used in these studies were enlisted and classified as independent FTD-focused scales and general psychopathology rating scales, the items of which were used to describe FTD in these studies. Further, we also included FTD-focused scales that were found by hand-searching.

All rating scales – irrespective of if they were originally produced in English – were included. To avoid redundancies in the general psychopathology rating scales, we further used only those scales that were used in more than 10 studies with patient samples to study FTD. This criterion was not applied to FTD-specific scales. Although the Bern Psychopathology Scale (BPS-R; Strik et al., 2010) has three domains (language, affectivity, and motor behavior), due to its rich and specific description of FTD components, we considered it as a FTD-specific scale based on the language domain. The tools that used only self-reporting questionnaires (Barrera, McKenna, & Berrios, 2008; McGrath & Allman, 2000; Miers & Raulin, 1987; Smirnova et al., 2020) or cognitive tests (Bannister & Fransella, 1966; Whitaker, 1978) to measure FTD were excluded. Scales that used conversational constraints but still tested specific cognitive deficits (e.g. social cognition (Bazin, Sarfati, Lefrère, Passerieux, & Hardy-Baylé, 2005)) or focused on specific interpersonal dynamics (e.g. at family level (Wynne & Singer, 1963)) were also excluded.

**Figure 1.**
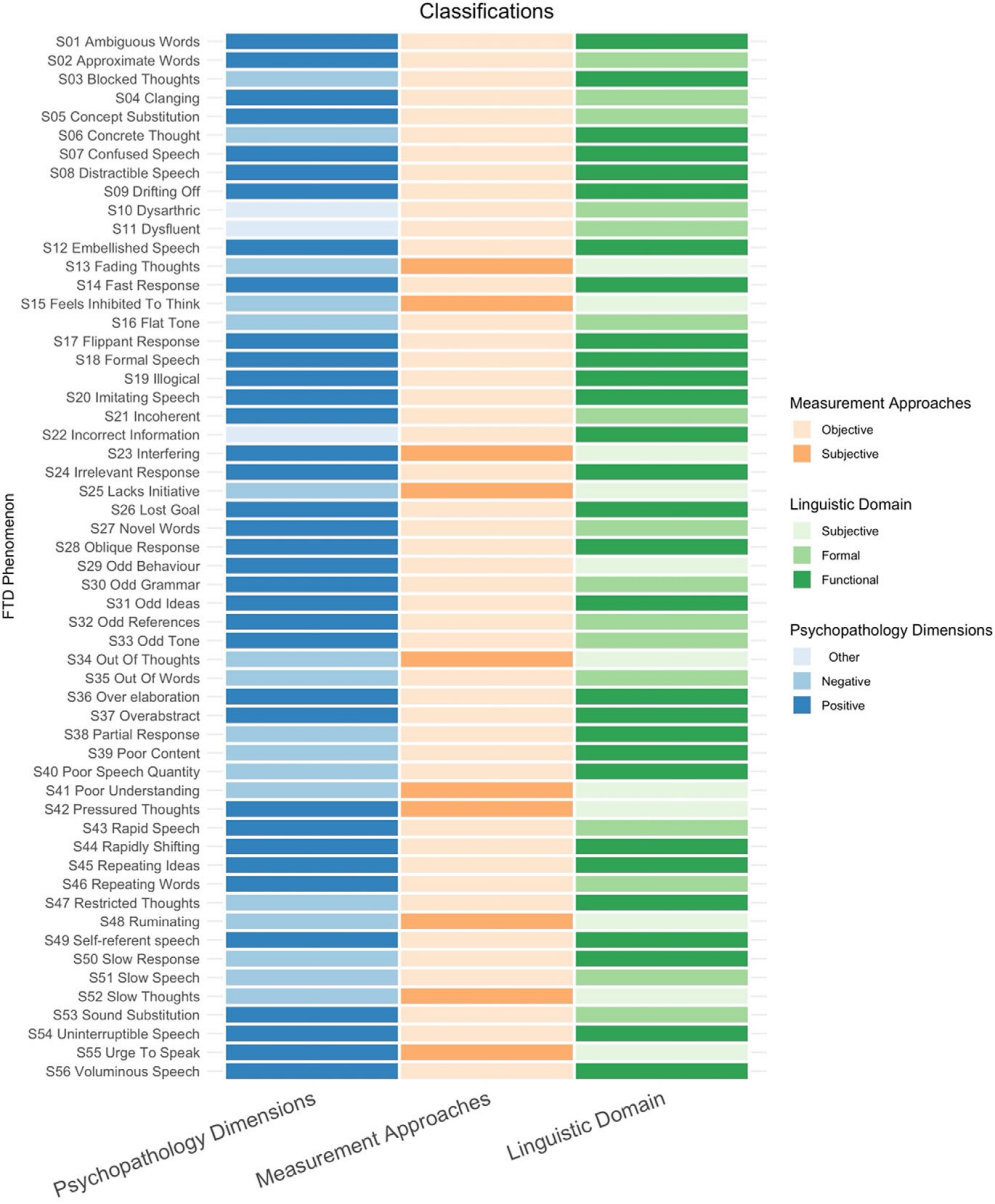
Classification of the identified FTD phenomena. Psychopathology dimensions include the traditional positive and negative features, with three features considered indeterminate (other). Measurement approaches refer to objective and subjective phenomena as rated in the FTD scales. Linguistic domain refers to formal and functional linguistic competence. Of note, subjective phenomena are not classified under linguistic domains.This list is alphabetically ordered.

Finally, we included 12 FTD-specific scales, and 4 general psychopathology scales for further analyses (Figure 1). The Scales for Assessment of Positive and Negative Symptoms (SAPS-SANS (Andreasen, 1990)) were considered as a single scale in further analyses, bringing the total to 15. Thus, to assess how rating scales for FTD map in terms of item content, the items from 15 scales, totaling 207 FTD items, were examined. The scales and the corresponding number of FTD items included from each scale are as follows: Bizarre idiosyncratic thinking - BIT (11 items (Harrow & Quinlan, 1985; Marengo, Harrow, Lanin-Kettering, & Wilson, 1986)), Brief Psychiatric Rating Scale Expanded - BPRS-E (8 items (Lukoff, Liberman, & Nuechterlein, 1986; Ventura et al., 1993)), Bern Psychopathology Scale Revised BPS-R (15 items (Strik et al., 2010)), Communication Deviance Index CDI (6 items (Docherty, DeRosa, & Andreasen, 1996)), Clinical language disorder rating scale CLANG (17 items (Chen et al., 1996)), Kiddie Formal Thought Disorder KFTD (4 items (Caplan, Guthrie, Fish, Tanguay, & David-Lando, 1989)), Loosening of Association and Disordered Speech patterns LADSP (10 items (Reilly, Harrow, Tucker, Quinlan, & Siegel, 1975)), Positive and Negative Syndrome Scale PANSS (9 items (Kay, Fiszbein, & Opler, 1987)), SAPS-SANS (13 items (Andreasen, 1990)), Thought and Language Disorder Scale TALD (30 items (Kircher et al., 2014)), Thought Disorder Index TDI (23 items (Johnston, 1979)), Thinking Dysfunction Rating Scale TDRS (22 items (Karasu, Plutchik, Nemetz, & Conte, 1979)), Operationalized Experimental Psychic Test TePEO (11 items (Foucher et al., 2018)), Thought, language, and communication scale TLC (20 items (Andreasen NC, 1979)), and Thought Language Index TLI (8 items (Liddle et al., 2002)).

### Extracting FTD phenomena

We defined an individual FTD ‘phenomenon of interest’ as a feature that is captured as an item by at least one of the 15 FTD scales and is distinguishable in meaning from other phenomena of interest. To identify each phenomenon of interest, we used a rubric formed by two scales: the scale with the most items (TALD) and the most commonly used FTD scale (TLC), and mapped the features identified from every other scale items onto this rubric. The core phenomena captured in these scales were initially listed as the first set, which was subsequently expanded as new phenomena were identified from other scales. The terminologies used in the FTD scales include a large number of metaphorical labels e.g. derailment and tangentiality. Furthermore, non-correspondence was noted across the same terms in different scales e.g. tangentiality in TLC aligns more closely with TALD’s cross-talk item, than TALD’s item of tangentiality per se (see Voppel and colleagues for a discussion of these discrepancies (Voppel et al., 2025)). As a result, we did not reuse any of the scale-based phenomenological terms, e.g. TLC tangentiality and TALD crosstalk were mapped onto ‘unrelated response’ in our set. For every scale we considered, items were mapped onto one or more phenomena. One-to-one mapping was either specific (a scale item description that fully matched a phenomenon of interest) or general (a scale item simply referred to a phenomenon, enabling its measurement in a broad sense). One item had only general and no specific mapping (‘embellished speech’); we made a consensus decision to include it given its distinct quality when compared to circumstantiality (Kelly & Casey, 2024). Some scales had single items encompassing multiple phenomena, such as the PANSS Conceptual Disorganization item, which captures tangentiality, derailment, incoherence, and illogicality all in a single item score. This feature of compounded measurement was very frequent in FTD compared to other domains where interchangeability has been studied to date (e.g. depression (Fried, 2017), clinical high risk (Bernardin et al., 2023), mental pain (Charvet et al., 2022), youth-onset depression (Vilar et al., 2024)). Consequently, we quantified complexity scores (i.e. the number of individual phenomena captured per item in the scale expressed as a percentage; values >1 indicate a high degree of compounding, while values <1 indicate redundancy with more than 1 item describing the same phenomena).

Several decisions were made when mapping the item pool from the included scales onto the list of phenomena. In the TDI (Johnston, 1979), Rorschach-specific cognitive scores (e.g. Fragmentation, Incongruous Combination, and Arbitrary Form-Color Response) were removed as they are not generalizable outside of the projective test context. The ‘delusional thinking’ item of the LADSP scale (Reilly et al., 1975) was removed to maintain a clear distinction between formal thought disorder and assessment of thought content alone. Each set of the five confabulation items of TDI, three illogicality items of BIT (Marengo et al., 1986), and two referential failure items of CDI (Docherty et al., 1996) were collapsed into a single symptom when mapping items to avoid overrepresentation. BPS-R (Strik et al., 2010) had items that were rated bidirectionally, i.e. with negative or positive scores on a spectrum, e.g. scoring of spontaneity ranged from reduced to increased. However, considering the possibility of the presence of both spectrum of symptoms in a given patient, we included these as discrete phenomena. LP, VSS, and GV discussed and iteratively derived the consensus list of FTD phenomena and their descriptions between January 2024 and November 2025, with examples derived from either original scales (TLC, TALD, and CDI) or newly written (in most cases).

To study the areas of coverage across scales, we classified individual phenomena at three levels: psychopathological dimensions (positive versus negative, informed by Fish (Fish, 1976), Strauss (Strauss, Carpenter, & Bartko, 1974), and Andreasen (Andreasen NC, 1979) applications of these terms), measurement approaches (subjective versus objective, based on Kircher’s application (Kircher et al., 2014)), and the linguistic domains for the objective phenomena (formal versus functional competence). For linguistic domains, we used the reframing of a long-standing tradition that differentiates between formal and functional linguistics (Chomsky, 1965), as provided by Mahowald and colleagues (Mahowald et al., 2024). We focused on phonology, lexico-semantic, and syntax for formal competence, and logical reasoning, pragmatics, world knowledge, and situation models for functional competence.

### Statistical analysis

Content overlap among the scales was estimated using a similarity coefficient called the Jaccard Similarity Index: J(A, B) = ∣A∩B∣/∣A∪B∣ in line with Fried and colleagues (Fried, 2017). We interpreted the resulting values based on Evan’s criteria (Evans, 1996) of very weak 0.00–0.19, weak 0.20–0.39, moderate 0.40–0.59, strong 0.60–0.79, and very strong 0.80–1.0. We calculated the JSI for each scale pair and computed an overall average for each scale and for FTD as a whole, as well as for each of the three levels of FTD phenomena (positive/negative, formal/functional and objective/subjective). For each scale pair, we also computed the total specific phenomena covered per pair (the coverage index) among the total enlisted symptoms, and rank-ordered them to assess the best possible combinations that achieve the best overall coverage. Finally, we evaluated the optimal multi-instrument combination with the fewest number of scales required to achieve the best possible coverage of FTD. To answer the question of whether the length of the scale is associated with a higher overlap of its symptoms with the other scales measured in this paper, we measured the correlation between the mean Jaccard coefficient of each scale and the length of the scale for the number of specific symptoms captured and adjusted for scale length (Fried, 2017). Analyses were conducted using Python script (https://mybinder.org/v2/gh/vincentpmartin/generic.content.analysis/HEAD?labpath=jupyter_notebook_generic_content_analysis.ipynb) and R software (R Core Team, 2021).

## Results

With respect to the question how many distinct features comprise the construct of FTD as currently measured, our approach identified a total of 56 discrete phenomena based on the content of 15 scales (Figure 1; See Appendix A for expanded descriptions, classification and examples). Among the 56 phenomena, 36 were identified as positive, 17 as negative and 3 were not describable as negative or positive phenomena. 10 subjective phenomena were noted. Of the 46 objective phenomena, 16 tapped on formal linguistic competence (7 phonology, 7 lexico-semantics, and 2 syntax), 29 tapped on functional linguistic competence (19 pragmatics, 2 world knowledge, 7 situation modeling, and 1 on logical reasoning); one (odd behavior) was not classifiable on the basis of linguistic domains (see Supplementary Figure S2).

### Overlap of FTD phenomena across scales

With respect to the question of the degree of overlap, each phenomenon was featured in a median of only 4 of 15 scales (mean [SD] of 4.86 [+/−2.86]) in a broad sense, and 3 scales in a stricter sense (mean [SD] of 3.52 [+/−2.52]). This indicates that only one-fifth of the scales captured the *same* FTD phenomenon on average.

**Figure 2.**
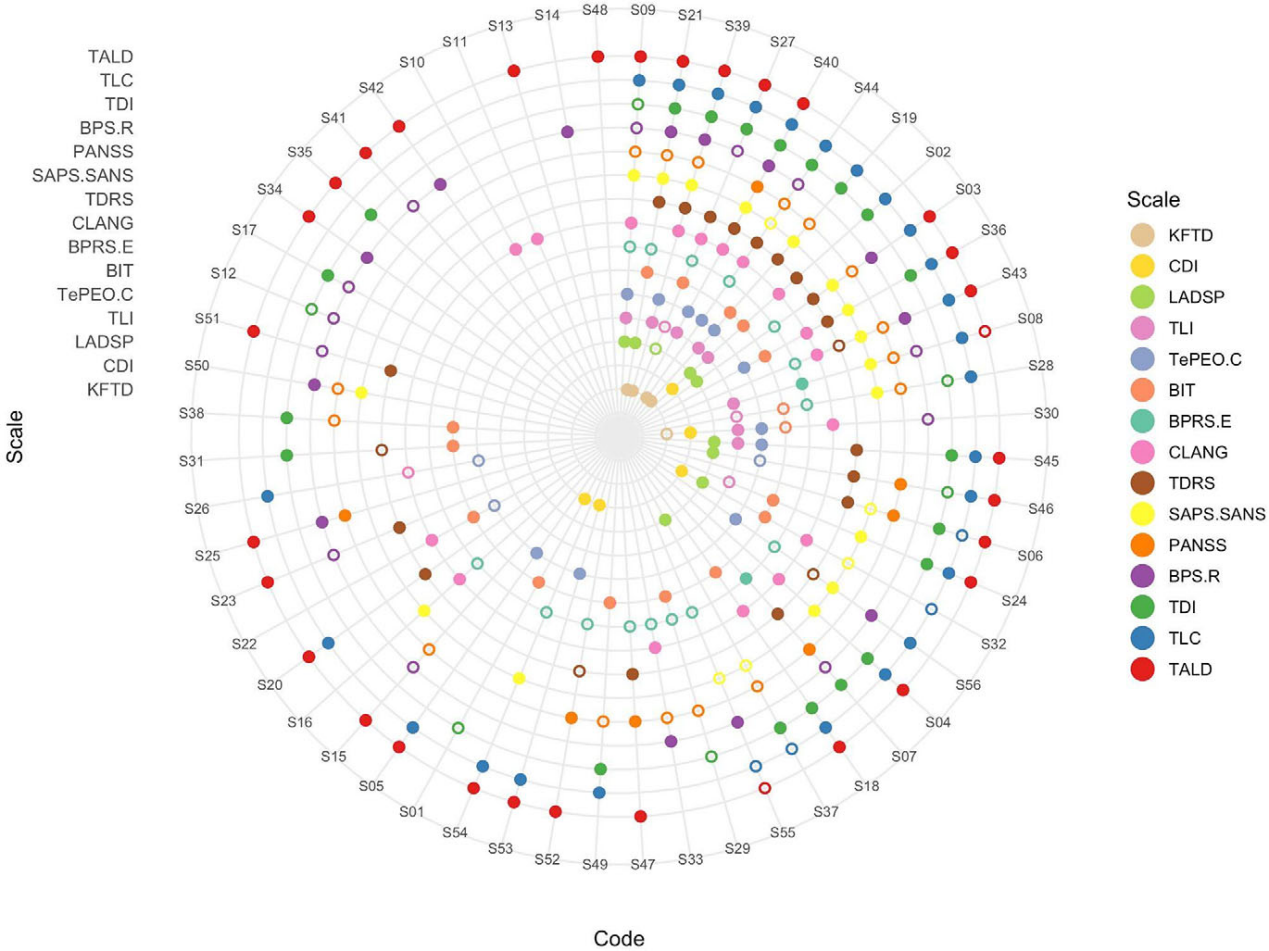
Radar wheel map showing the frequency of individual FTD phenomena across the 15 scales. *Note*: Each circle represents an individual scale. Each spoke represents one of the 56 FTD phenomena. Color filled dots at their intersection indicate the phenomena being captured specifically by each scale; Open circles represent the phenomena captured generally (i.e. in a broad sense) by each scale. S01, Ambiguous Words; S02, Approximate Words; S03, Blocked Thoughts; S04, Clang Words; S05, Concept Substitution; S06, Concrete Thought; S07, Confused Speech; S08, Distractible Speech; S09, Drifting Off; S10, Dysarthric; S11, Dysfluent; S12, Embellished Speech; S13, Fading Thoughts; S14, Fast Response; S15, Feels Inhibited To Think; S16, Flat Tone; S17, Flippant Response; S18, Formal Speech; S19, Illogical; S20, Imitating Speech; S21, Incoherent; S22, Incorrect Information; S23, Interfering; S24, Irrelevant Response; S25, Lacks Initiative; S26, Lost Goal; S27, Novel Words; S28, Oblique Response; S29, Odd Behavior; S30, Odd Grammar; S31, Odd Ideas; S32, Odd Reference; S33, Odd Tone; S34, Out Of Thoughts; S35, Out Of Words; S36, Overelaboration; S37, Overabstract; S38, Partial Response; S39, Poor Content; S40, Poor Speech Quantity; S41, Poor Understanding; S42, Pressured Thoughts; S43, Rapid Speech; S44, Rapidly Shifting; S45, Repeating Ideas; S46, Repeating Words; S47, Restricted Thoughts; S48, Ruminating; S49, Self-referent speech; S50, Slow Response; S52, Slow Speech; S51, Slow Thoughts; S53, Sound Substitution; S54, Uninterruptible Speech; S55, Urge To Speak; S56, Voluminous Speech; TALD, Thought and Language Disorder Scale; TLC, Thought, Language, and Communication scale; TDI, Thought Disorder Index; BPS.R, Bern Psychopathology Scale Revised; PANSS, Positive and Negative Syndrome Scale; SAPS.SANS, Scales for assessment of positive and negative symptoms; TDRS, Thinking Dysfunction Rating Scale; CLANG, Clinical language disorder rating scale; BPRS-E, Brief Psychiatric Rating Scale Expanded; BIT, Bizarre idiosyncratic thinking; TEPEO.C, Operationalized Experimental Psychic Test; TLI, Thought Language Index; LADSP, Loosening of Association and Disordered Speech patterns; CDI, Communication Deviance Index; KFTD, Kiddie Formal Thought Disorder.

Among the group of 56 phenomena, none were featured in all of the scales; 3 were seen in 11 of the 15 scales (‘drifting off’, ‘incoherent’, and ‘poor content of speech’; Figure 2) in a broad sense, but when specific mapping were considered, even these top three phenomena were covered only in 7 (i.e. <50%) scales. In total, 20% of FTD phenomena (11 of 56) can be termed *idiosyncratic* (e.g. ‘dysarthric’, ‘dysfluent’, ‘fading thoughts’, ‘fast response’, ‘ruminating’, etc.) being featured specifically in only one of the 15 scales (Table 1). Only 11 items (<20% of the total) are covered by 8 or more scales (>50% of scales).

**Table 1.** Number of FTD phenomena featured across scales

Number of scales	Number/% of phenomena featured (broad)	Number/% of phenomena featured (specific)
>12	0 (0)	0 (0)
11	3 (5)	0 (0)
10	3 (5)	2 (4)
9	1 (2)	1 (2)
8	4 (7)	3 (5)
7	5 (9)	2 (4)
6	5 (9)	3 (5)
5	4 (7)	5 (9)
4	7 (13)	7 (13)
3	13 (23)	6 (11)
2	6 (11)	15 (27)
1	5 (9)	11 (20)

### Content coverage and interchangeability

TALD had the highest coverage (54% specific; 57% general) while KFTD had the lowest general coverage (7% specific; 9% general; Table 2). In general, scales predominantly covered positive (average of 76.1% of items across the scales; 56.2–100% of individuals scales) and functional FTD phenomena (60.5% of items on average across the scales, 20–76.2% of individual scales). In particular, pragmatic phenomena had the maximal coverage among the subdomains (35.15% on average; 11–52.4% of individual scales. See Supplementary Figure S3). As expected, complexity was high for multi-domain scales such as PANSS and BPRS-E (>2 phenomena per item on average; Table 2) and consequently their specific coverage was low (4% for BPRS-E and 11% for PANSS).

**Table 2.** Coverage and complexity of individual FTD scales

Instrument	Original items	Phenomena (Broad)	Phenomena (Specific)	Total phenomena	General Coverage	Complexity	Specific Coverage
BIT	11	2	14	16	29%	1.45	25%
BPRS-E	8	15	2	17	30%	2.13	4%
BPS-R	15	11	14	25	45%	1.67	25%
CDI	6	0	5	5	9%	0.83	9%
CLANG	17	1	17	18	32%	1.06	30%
KFTD	4	1	4	5	9%	1.25	7%
LADSP	10	1	8	9	16%	0.90	14%
PANSS	9	17	6	23	41%	2.56	11%
SAPS-SANS	13	5	16	21	38%	1.62	29%
TALD	30	2	30	32	57%	1.07	54%
TDI	23	6	20	26	46%	1.13	36%
TDRS	22	4	17	21	38%	0.95	30%
TePEO-C	11	3	11	14	25%	1.27	20%
TLC	20	4	25	29	52%	1.45	45%
TLI	8	3	8	11	20%	1.38	14%

BIT, Bizarre idiosyncratic thinking; BPRS-E, Brief Psychiatric Rating Scale Expanded; BPS.R, Bern Psychopathology Scale Revised; CDI, Communication Deviance Index; CLANG, Clinical language disorder rating scale; KFTD, Kiddie Formal Thought Disorder; LADSP, Loosening of Association and Disordered Speech patterns; PANSS, Positive and Negative Syndrome Scale; SAPS.SANS, Scales for assessment of positive and negative symptoms; TALD, Thought and Language Disorder Scale; TDI, Thought Disorder Index; TDRS, Thinking Dysfunction Rating Scale; TEPEO.C, Operationalized Experimental Psychic Test; TLC, Thought, language, and communication scale; TLI, thought Language Index.

**Figure 3.**
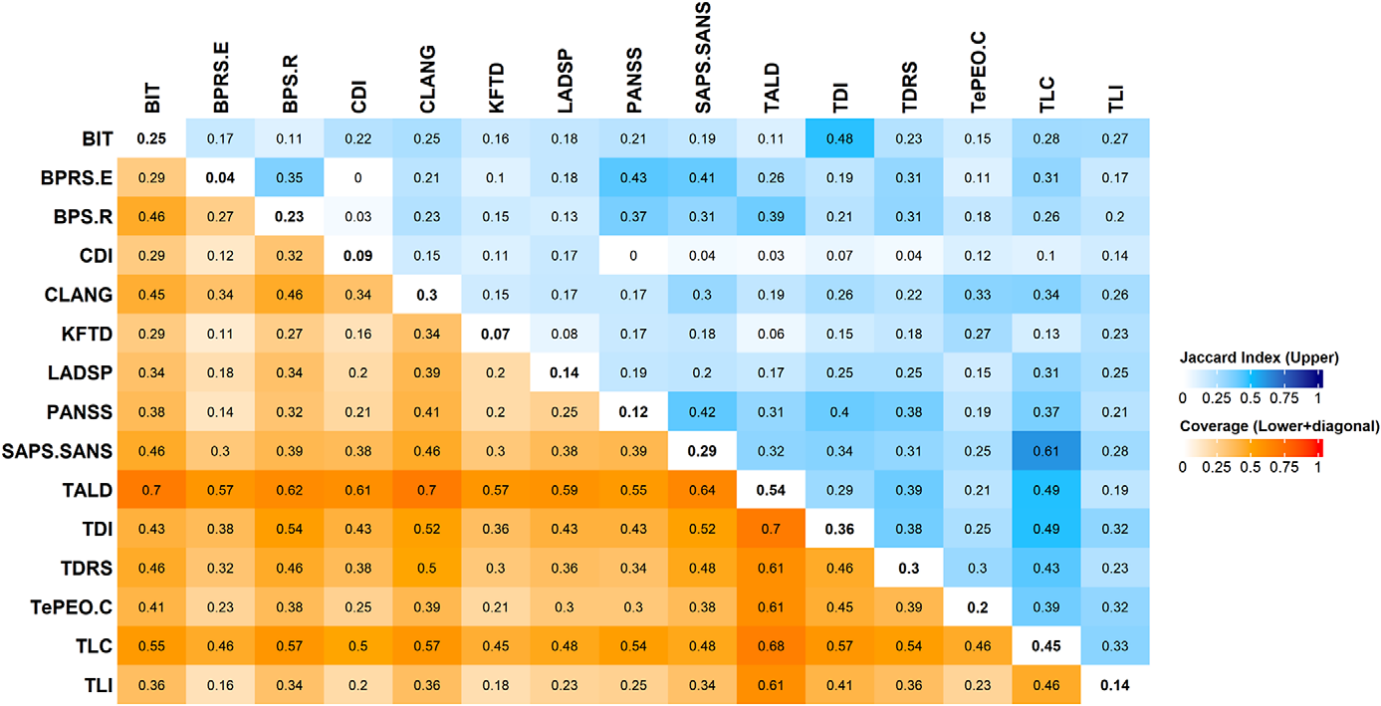
Jaccard similarity map and specific coverage map. The bottom half indicates specific coverage achieved by each scale when used in combination with another scale. The diagonal indicates the overall coverage of specific phenomena (out of 56) achieved by each scale. The upper half represents the Jaccard Similarity Index between any given pair of scales. See main text for expansions of the acronyms denoting each scale.

Based on 105 individual Jaccard Similarity Index scores (one per pair of scales) the maximum score was 0.35 (TLC) and the minimum was 0.09 (CDI), with the average being 0.24 across the FTD measurements (Figure 3). Using Evan’s criteria, FTD scales can be said to have weak or very weak similarity among each other and thus not interchangeable in their utility. Certain pairs of scales overlap more with each other than others, e.g. 0.61 for TLC-SANS/SAPS (developed by the same author) followed by scales developed with similar theoretical assumptions (0.49 for TLC-TALD; both based on historical terms in psychopathology; 0.48 for TDI-BIT, both developed from projective tests). But several pairs of scales (e.g. BPRS-E and CDI) had near-zero overlap, weakening any claim that they measure the same construct.

The top pair of scales providing maximum coverage of specific items (71%) was a combination of BIT and TALD, followed by either CLANG, TDI, or TLC with TALD (70%; Supplementary Table S1). To achieve maximum and specific coverage (98%), a minimum of six scales had to be combined (TALD, TLC, CLANG, BIT, BPS-R, and TDI), a feat that is not practical for routine deployment. Combining scales with TLC improves the coverage of positive FTD, while TALD improves negative FTD coverage (Supplementary Figure S4).

## Discussion

This systematic item-level analysis reveals three critical findings regarding the measurement of FTD in psychosis: (1) At least 56 distinct phenomena make up the construct of FTD as measured using rating scales; (2) only a few of these features are shared among existing instruments, with no single sign/symptom being common to all 15 scales and nearly one-fifth of all identified phenomena being measured in only 1 of 15 scales; (3) weak overall content overlap indicates poor interchangeability among scales measuring FTD. Taken together, these observations challenge the notion that any rating scale is currently assessing the full ‘universe’ of FTD. These results offer a crucial step toward improving the replicability and generalizability of findings related to mechanistic and interventional studies in FTD.

Measurement discordance has direct and detrimental implications for both research and clinical practice (Uher, 2023). Poor interchangeability of scales hinders the synthesis of data across mechanistic and interventional studies, thereby impeding meta-analyses and the replication of findings essential for scientific progress (Fried, Flake, & Robinaugh, 2022). For instance, a treatment reported as effective for FTD using one scale may have targeted a specific set of phenomena (e.g. pragmatic deficits) that are not adequately captured by another scale used in a follow-up trial, leading to apparent failures in replication. Clinically, the reliance on disparate scales complicates the consistent monitoring of symptoms over time. FTD phenomena are increasingly recognized as valuable indicators of psychosis onset (Corcoran et al., 2018) and relapse (Dalal et al., 2026; Zaher et al., 2024), in addition to their role in prognostication across severe mental illnesses (Kircher et al., 2018; Palaniyappan & Wang, 2025). Despite this promise, measurement fragmentation ultimately contributes to the continued neglect of FTD as a therapeutic target, as the field lacks a unified metric to reliably detect change or predict outcomes like relapse and functional impairment.

Our analysis raises the question of the source of heterogeneity among FTD scales. There is a large diversity of clinical and theoretical assumptions regarding what constitutes disorganized thinking across various time periods of scale development (Barrera, 2025; Jerónimo, Queirós, Cheniaux, & Telles-Correia, 2018). These assumptions have heavily influenced item choices. The TLC scale, for instance, follows the clinical-descriptive tradition focusing on observable speech anomalies. At the same time, the TDI delves into the inferred cognitive processes behind these anomalies, rooted in a more psychoanalytic framework. For scales derived from general psychopathology instruments such as the PANSS or BPRS-E, FTD is often condensed into a single, comprehensive conceptual disorganization item, reflecting a prioritization of broad symptom coverage over specific phenomena of interest. Some of the differences also stem from the varied purposes for which these scales were developed. Some scales, like the KFTD (Caplan et al., 1989), were designed for brevity and rapid clinical assessment in children, while others, like the TALD (Kircher et al., 2014), were constructed to provide a comprehensive, fine-grained analysis for detailed psychopathological research. This, in turn, differs from the approach of scales like the BPS-R, which aims to capture the flow of experience by rating language phenomena on a bipolar aspect (e.g. from reduced to increased spontaneity) (Strik et al., 2010).

Several authors have reported high correlation between their ‘new’ scales and other existing instruments (e.g. TLC and TLI, TLC and TALD). Using depression as an example, Fried convincingly argues that even if there is a high degree of correlation in total summed scores across different instruments, this does not imply they measure the same construct (Fried et al., 2022). This is because such correlations emerge as a function of scale length and the average inter-item correlation. It is statistically possible for two scales with completely non-overlapping item content, e.g. one measuring phonological features and syntax, the other measuring pragmatics and logical reasoning, to produce moderately correlated sum-scores if their internal structures are similar. This statistical illusion of agreement masks the fact that these scales are capturing fundamentally different aspects of FTD. This issue is further exacerbated by the fact that FTD phenomena are not *exchangeable*, i.e. we cannot infer positive phenomena by measuring only the negative phenomena, or functional linguistic competence by studying only the formal competence. The different FTD features are likely to have distinct neural correlates and clinical trajectories, and are thus not merely proxies for a single, unified construct. Thus, the weak content overlap revealed by the low Jaccard Similarity Index represents a fundamental threat to construct validity, one that cannot be resolved by appealing to correlations between total scores.

The findings of this study should be interpreted in light of certain limitations. While our systematic scale selection aimed for comprehensiveness, the exclusion of self-report or less commonly used instruments means that the full spectrum of measured FTD phenomena may not be entirely captured; however, this is unlikely to alter the core finding of weak overlap. Furthermore, the consensus-driven mapping of scale items to discrete phenomena, though conducted by clinical experts, involved interpretive judgments, particularly for compounded items. Finally, our analysis focused exclusively on content validity and did not assess other psychometric properties such as reliability or convergent validity. However, the demonstrated conceptual heterogeneity presents a fundamental challenge to the theory of measurement that cannot be resolved by psychometric properties alone.

In conclusion, we provide the first empirical census of FTD phenomena, highlighting the urgent need for a standardized measure. Using six scales for complete coverage is clinically impractical. One solution is to harmonize existing scales using the landscape of shared constituents. We can also build a core measurement set from the foundation provided by the 56 phenomena identified here (see Assessment of Thought, Language, And Speech ATLAS checklist in Supplementary Materials). Given the exhaustive nature of the current list of items, it is feasible to generate machine-generated ratings of recorded speech transcripts based on this universe of FTD phenomena (see Supplementary Figure S5 for a worked-out example using DeepSeek-R1; Guo et al., 2025). With professional and experiential expert-based input, we can validate content and prioritize items, creating a consensus-based framework. This will harmonize our tools and ensure future neurobiological and treatment research is replicable and coherent. Improving measurement is a critical next step to ensure that future research into FTD is ultimately effective in addressing this core and debilitating dimension of psychosis.

## Supporting information

10.1017/S0033291726104152.sm001 Sreeraj et al. supplementary material Sreeraj et al. supplementary material

## Supplementary material

The supplementary material for this article can be found at http://doi.org/10.1017/S0033291726104152.

## A. Appendix

1. **Ambiguous Words** (Objective, Positive, Functional: Pragmatics). This involves using words in a way meaning is not obvious in the given context. Patient: ‘I need to find the *button* of my life.’ Clinician: ‘What do you mean by button of your life? Patient: ‘Button is that thing that controls you, dictates your life, like in a remote.’
2. **Approximate Words** (Objective, Positive, Formal: Lexical Semantics). This involves using words in a way not commonly used (but meaning can be inferred from the context). ‘All my letters were written with a blue *paperskate*’ (approximating to a pen or pencil).
3. **Blocking** (Objective, Negative, Functional: Situation Modeling). This is an observable interruption of an ongoing line of thought. ‘I’m feeling fine. My mood is much better because I’ve…… hmm….’ (stops or pauses).
4. **Clanging** (Objective, Positive, Formal: Lexical Semantics). This denotes associating ideas based on sounds - rhymes, puns, etc. ‘I’m trying to make sense. I’m not making *cents.* I have to make dollars.’
5. **Concept Substitution** (Objective, Positive, Formal: Lexical Semantics). This describes substituting one word with a conceptually related word. ‘She got a new. Outfit thing. for her wedding.’ (substituting for dress)
6. **Concrete Thought** (Objective, Negative, Functional: Pragmatics). This refers to interpreting only the concrete meaning of a statement e.g. a proverb. ‘What does it mean to say ‘Too many cooks spoil the broth’? - ‘Well… each cook has his or her own recipe, do not they. They cannot make the same broth.’
7. **Confused Speech** (Objective, Positive, Functional: Situation Modeling). This denotes being unsure/lacking certainty, generally about the current context (time, place, person). ‘I… I think. I am not sure if this is 1920 or 2020. Hmm. 1920 makes more sense because., I cannot picture the year. Is it before or after the pandemic? I am not certain.’
8. **Distractible Speech** (Objective, Positive, Functional: Situation Modeling). This is when the flow of speech is distracted by irrelevant external stimuli. Clinician: ‘Can you tell me about your job?’ Patient: ‘Well, it’s not anything big, I am just a person who puts files away - oh, that’s a bright red car outside. I used to have a red bike. Not anymore.’
9. **Drifting Off** (Objective, Positive, Functional: Situation Modeling). This is when ideas gradually move away from the starting point. Clinician: ‘Can you tell me about your job?.’ Patient: ‘I work in an office, can’t do it from home because I need to put files away everyday. It’s a tall building with lots of windows. Yeah, a good place. The large windows, they let in a lot of light… important for all those plants there.’
10. **Dysarthric** (Objective, Formal: Phonology). This refers to articulation difficulties. ‘My moup feels heaby’ (My mouth feels heavy).
11. **Dysfluent** (Objective, Formal: Phonology). This refers to stuttering, false starts, hesitations. ‘I was… I was going to… um… the thing is, I mean, I intended to… to make the call.’
12. **Embellished Speech** (Objective, Positive, Functional: Pragmatics). This describes excessive liveliness and descriptions in speech. Clinician: ‘Can you tell me about your job?’ Patient: ‘Ah, my job! I am not merely a filing clerk; I am the grand archivist of a vast, paper-based empire. Each document is a unique character in an unfolding epic, and my desk is the stage upon which the great drama of corporate life plays out. I don’t just sort papers; I orchestrate a symphony of information, weaving the mundane threads of invoices and memos into a vibrant tapestry of bureaucratic wonder!.’
13. **Fading Thoughts** (Subjective, Negative, Other: Subjective). This is the experience of the disappearance of ongoing thoughts. ‘When I watch TV, it seems like my thoughts gradually fade away.’
14. **Fast Response** (Objective, Positive, Functional: Pragmatics). Reduced latency in responding to a question or taking a conversational turn.
15. **Feels Inhibited To Think** (Subjective, Negative, Other: Subjective). This is the experience of resistance to thinking. ‘Thoughts don’t flow anymore. It is hard work to continue thinking on any subject.’
16. **Flat Tone** (Objective, Negative, Formal: Phonology). This is speech that lacks intonation and emotional quality; flat and monotonous speech. (audio).
17. **Flippant Response** (Objective, Positive, Functional: Pragmatics). This involves joking responses that lack seriousness and do not convey the expected answers. Clinician: ‘Can you tell me about your job?’ Patient: ‘Oh, I’m a professional Jedi Master of the photocopier. My main skill is translating coffee into productivity.’
18. **Formal Speech** (Objective, Positive, Functional: Pragmatics). This describes an old-fashioned, archaic, and stilted style of speech. Clinician: ‘Can you tell me about your job?’ Patient: ‘I am engaged in the execution of administrative duties and the management of correspondence within a commercial enterprise.’ (instead of saying ‘I work as an office clerk, mostly handling paperwork’)
19. **Illogical** (Objective, Positive, Functional: Formal reasoning). This is characterised by drawing unwarranted conclusions not logically preceded by appropriate premises. Clinician: ‘Can you tell me about your family?’ Patient: ‘Parents are the people that raise you. Anything that raises you can be a parent. Parents can be anything, material, vegetable, or mineral, that has taught you something. Parents would be the world of things that are alive, that are there. Rocks, a person can look at a rock and learn something from it, so that would be a parent.’ (This example is from Andreasen’s TLC scale)
20. **Imitating Speech** (Objective, Positive, Functional: Pragmatics). This is when the patient echoes the words or phrases of the interviewer. Clinician: ‘Can you tell me about your job?’ Patient: ‘about your job… about your job….’
21. **Incoherent** (Objective, Positive, Formal: Syntax). This is when words are put together in a sentence in a way that fails to convey meaning. Clinician: ‘Can you tell me about your job?’ Patient: ‘I don’t work now, often the circular greenness of the potato sings elbows under the transparent highway.’
22. **Incorrect Information** (Objective, Other, Functional: World Knowledge). This refers to providing incorrect or false factual information. Clinician: ‘Why do you think some people believe in God?’ Patient: ‘People believe in a religion. Anything is a religion. God is just the center of the religion. Europe is a religion, and *Washington*, *being the capital*, is God.’ (This example is from Chen’s CLANG scale)
23. **Interfering thoughts** (Subjective, Positive, Other: Subjective). This is the experience of intruding thoughts that do not belong to the current line of thinking. ‘My ability to concentrate is gone. It is easy to distract me.’ (This example is from Kircher’s TALD)
24. **Irrelevant Response** (Objective, Positive, Functional: Pragmatics). This refers to providing a response that is not connected to the question posed. Clinician: ‘Can you tell me about your job?’ Patient: ‘The moon was full last night. It looked like a silver coin.’
25. **Lacks Initiative** (Subjective, Negative, Other: Subjective). This is the experience of difficulty initiating thoughts intentionally. ‘I don’t have the strength to take up something that needs thinking and concentration.’
26. **Lost Goal** (Objective, Positive, Functional: Situation Modeling). This is when speech moves from the subject and never returns to it. Clinician: ‘Can you tell me about your job?’ Patient: ‘My job? Not much to say about it. It is a job downtown. Not easy getting to downtown these days… with all these school buses every morning. You know, there is a new school downtown.’
27. **Novel Words** (Objective, Positive, Formal: Lexical Semantics). This refers to inventing new words for concepts. ‘I left the papers on the *deskilator.*’
28. **Oblique Response** (Objective, Positive, Functional: Pragmatics). This involves providing a response that is inexact or vaguely related. Clinician: ‘Can you tell me about your job?’ Patient: ‘A job is a thing that requires a lot of responsibility. Responsibility is a heavy weight. How much weight can one take these days, eh?.’
29. **Odd Behavior** (Objective, Positive, Other). This describes unusual behaviors e.g. self-talking, self-smiling, and so forth ‘Clinician: “Can you tell me about your job?’ Patient: (leans forward and whispers to their own hand).
30. **Odd Grammar** (Objective, Positive, Formal: Syntax). This describes the use of an abnormal sentence construction (syntax). Clinician: ‘Can you tell me about your job?’ Patient: ‘The job, the filing is it all about it, it is done by me, with the papers, in the room, the same one.’
31. **Odd Ideas** (Objective, Positive, Functional: World Knowledge). These are ideas that defy ordinary social conventions. Clinician: ‘Can you tell me about your job?’ Patient: ‘My job’s purpose is to prepare packages of papers. All of them carry the required ink. I keep the knowledge of the addresses they go to reside in’ (filing job at an office).
32. **Odd References** (Objective, Positive, Formal: Lexical Semantics). This describes unclear links to people, objects, and so forth when using pronouns, articles. Clinician: ‘Can you tell me about your job?’ Patient: ‘I am a clerk. Well, they are always putting it over there with the thing, and then she doesn’t know what to do with them. I sort all of that out, all day.’
33. **Odd Tone** (Objective, Positive, Formal: Phonology). This is a bizarre quality of voice e.g. high-pitch, mechanical, very loud, very soft etc. (audio).
34. **Out Of Thoughts** (Subjective, Negative, Other: Subjective). This is the feeling as if there are no thoughts/ideas available to express. ‘I… I can’t think much. There’s nothing there. It’s just blank.’
35. **Out Of Words** (Objective, Negative, Formal: Lexical Semantics). This is when a patient finds it difficult to bring up the correct word for a concept. Clinician: ‘Can you tell me about your job?’ Patient: ‘I am a clerk. Most of the time I work with the… the… you know, the big machine that copies everything… not an X-ray… the one that multiplies papers! No, that’s not it… the… ugh, I can’t get the word’ (photocopier).
36. **Over elaboration** (Objective, Positive, Functional: Pragmatics). This is a tedious, long-winded narrative that is delayed in making the point. Clinician: ‘Can you tell me about your job?’ Patient: ‘Well, to understand my job, you must first appreciate the fundamental nature of organizational structures in a post-industrial service economy. My particular role emerged from a corporate restructuring in Q2 of last year, which itself was precipitated by market forces… [continues for 2 minutes] …and after considering all these contextual factors, my position essentially involves files storage and data entry.’
37. **Overabstract** (Objective, Positive, Functional: Pragmatics). This involves speaking too generally; overinclusive without concrete boundaries. Clinician: What is similar about an orange-banana? Patient: They both contain atoms. (This example is from Johnston & Holzman’s TDI)
38. **Partial Response** (Objective, Negative, Functional: Pragmatics). This is responding as if only part of a question has been understood. Clinician: ‘Can you tell me about your job?’ Patient: ‘Yes. I can tell you.’ (Then remains silent, looking for the next question).
39. **Poor Content** (Objective, Negative, Functional: Pragmatics). This is when speech content lacks sufficient ideas despite being of a sufficient amount. Clinician: ‘Can you tell me about your job?’ Patient: ‘It’s a thing you do. You go there. You do the work that is there to be done. It’s fine. It’s a job. You know how jobs are. It’s one of them.’
40. **Poor Speech Quantity** (Objective, Negative, Functional: Pragmatics). This is when there is not a sufficient amount of words spoken in a given context. Clinician: ‘Can you tell me about your job?’ Patient: ‘Clerk’ (After a few seconds) Clinician: ‘can you tell me more?.’ Patient: ‘Nope. That’s it.’
41. **Poor Understanding** (Subjective, Negative, Other: Subjective). This is when a patient cannot readily understand the meaning of words or sentences. ‘Sometimes I feel like I’m not catching the meanings of the words I’m hearing.’
42. **Pressured Thoughts** (Subjective, Positive, Other: Subjective). This is the experience of too many thoughts that one cannot control or suppress. ‘The thoughts whirl around and around; there are too many of them.’
43. **Rapid Speech** (Objective, Positive, Functional: Pragmatics). This is when the rate of speech production is high. (audio).
44. **Rapidly Shifting** (Objective, Positive, Functional: Situation Modeling). This is the rapid shifting of ideas that are only connected weakly. Clinician: ‘Can you tell me about your job?.’ Patient: ‘I’m a cashier. it’s all about the numbers, numbers everywhere, like in a bingo hall, bingo makes me think of my grandmother, she used to cook the best stew, stew reminds me of winter, winter has the best skiing, I’ve never been skiing but I love the mountains, mountains look like giant scoops of ice cream… vanilla ice cream….’
45. **Repeating Ideas** (Objective, Positive, Functional: Situation Modeling). This is when the same ideas or themes recur within a narrative. Clinician: ‘Can you tell me about your job?’ Patient: ‘I work with AI. The AI are everything these days. I have to make sure the AI programs are running. Before this, I had a job with different AI system. AI programs need maintenance. Most jobs now cannot do without AI.’
46. **Repeating Words** (Objective, Positive, Formal: Lexical Semantics). This denotes using the same word repeatedly without different meaning. Clinician: ‘Can you tell me about your job?.’ Patient: “I am a philosopher, philosopher, I am a philosopher, who composed great literature. I have a calling for this work, a calling, calling. You don’t have to believe me, believe me, not believe, not believe.’
47. **Restricted Thoughts** (Objective, Negative, Functional: Pragmatics). This is when the range of content is restricted to a few topics with limited switching. Clinician: ‘Can you tell me about your job?.’ Patient: ‘Well, it’s hard to focus on my job because of this terrible backache. The pain is always there.’ Clinician: ‘What are your main tasks that you have to focus on?.’ Patient: ‘I suppose I have to sit at a desk and do the accounts, and that just makes the back pain worse. I’ve had it since I moved to this city.’ Clinician: ‘In see. When did you move here?.’ Patient: ‘It’s not long ago but soon after I found out that the office chairs aren’t very good. This back pain really makes it difficult to get around doing anything else.’
48. **Ruminating** (Subjective, Negative, Other: Subjective). This is the experience of constant preoccupation with mostly unpleasant topics. ‘I can’t stop thinking about that mistake I made last week. It plays over and over in my mind. I keep analyzing every detail, what I should have said, what I should have done. It’s exhausting but I can’t switch it off.’
49. **Self-referent Speech** (Objective, Positive, Functional: Pragmatics). This involves repeatedly referring the subject under discussion back to oneself. Clinician: ‘Can you tell me about your job?’ Patient: ‘My job is important to me. It shows who I am. When I work, I feel good about myself. It kind of gives me myself.’
50. **Slow Response** (Objective, Negative, Functional: Pragmatics). This is increased latency in responding to a question or taking a conversational turn. (audio).
51. **Slow Speech** (Objective, Negative, Functional: Pragmatics). This is when the rate of speech production is low. (audio).
52. **Slow Thoughts** (Subjective, Negative). This is the feeling of slowing down of thoughts. ‘My thoughts take a lot of time to move along.’
53. **Sound Substitution** (Objective, Positive, Formal: Lexical Semantics). This involves mispronunciations of words. Clinician: ‘Can you tell me about your job?’ Patient: ‘I’m a tepper… no, a typer… I mean, a typist.’
54. **Uninterruptible Speech** (Objective, Positive, Functional: Pragmatics). This is when it is difficult to interrupt the speech of a patient by the interviewer. (audio).
55. **Urge To Speak** (Subjective, Positive, Other: Subjective). This is the feeling of a strong drive or impulse to speak. ‘I have such a hard time thinking before I speak. I blurt out quite a lot.’
56. **Voluminous Speech** (Objective, Positive, Functional: Pragmatics). This is when the quantity of speech is increased. Clinician: ‘Can you tell me about your job?’ Patient: ‘I work in a store that sells things for your home. Every morning, I say hello to people. I ask what they need. I show them where things are. I explain what things do. I work mostly at the cash register. I put things back on the shelves. You will be shocked to know how I got this job. I…’ (keeps talking a lot, for a long time).
57. *Note:* The use of (audio) refers to the need for recorded acoustic information in order to identify a phenomenon. For a ready to use checklist of these 56 features mapped on to the rubric of a Mental State Examination, see Supplementary Material (Assessment of Thought, Language, And Speech ATLAS checklist).
