# Supplementary material for: What is being measured by formal thought disorder scales? An item-level content analysis
Source: Psychol Med. 2026 Apr 13;56:e106. doi: 10.1017/S0033291726104152 (PMC13079215; doi:10.1017/S0033291726104152)
Supplement: Sreeraj et al. supplementary material [file S0033291726104152sup001.pdf]

Supplementary figure S1: PRISMA Flowchart: 2
Supplementary Table S1: Excluded general psychopathology rating scales (due to citations <10 for FTD) 3
Supplementary Table S2: Included items from general psychopathology rating scales 4
Supplementary figure S2: Sunburst Map showing the classification of the FTD phenomena. 6
Supplementary figure S3: Heat maps of coverage of different domains of FTD phenomena by each FTD scale. 7
Supplementary Figure S4 8
Supplementary figure S5: Idiographic radar charts of FTD profiles based on speech transcripts 9
Supplementary Table S3: Top combinations of scales that complement each other to achieve the best coverage of the FTD phenomenon 10
Supplementary Material SM1: Mapping Linguistic Domains with FTD 11
Supplementary Material SM2 12

Clinical Assessment of Thought Language And Speech [ATLAS Checklist] 12

Supplementary figure S1: PRISMA Flowchart:

The flow chart represents the literature search, inclusion and exclusion decisions. Tools refer to FTD instruments/scales sought for this analysis. FTD= Formal Thought Disorder.

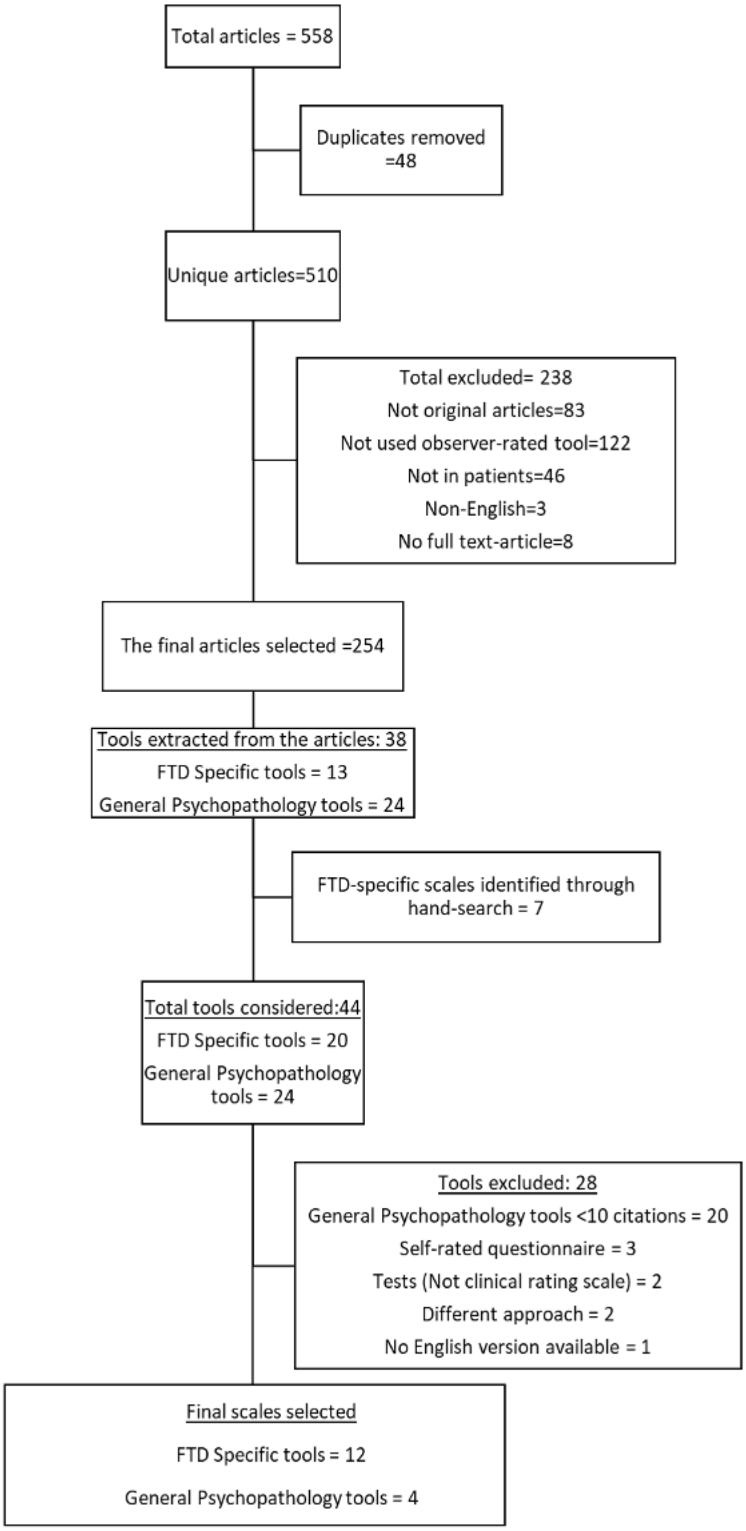

Supplementary Table S1: Excluded general psychopathology rating scales (due to citations <10 for FTD)

Scale / Assessment Tool	Acronym	Primary Focus / Clinical Use
**Young Mania Rating Scale**	YMRS	Assessment of manic symptoms in bipolar disorder.
**Hamilton Depression Rating Scale**	HAM-D	Measures the severity of depressive symptoms in adults.
**Montgomery-Åsberg Depression Rating Scale**	MADRS	Highly sensitive measure of changes in depression severity.
**Lifetime Dimensions of Psychosis Scale**	LDPS	Evaluates the presence and severity of psychosis over a lifetime.
**Structured Interview for Psychosis-Risk Syndromes**	SIPS	Used to identify individuals at clinical high risk for psychosis.
**Kiddie Schedule for Affective Disorders and Schizophrenia**	K-SADS	Semi-structured diagnostic interview for children and adolescents.
**Comprehensive Assessment of Symptoms and History**	CASH	Detailed instrument for documenting signs, symptoms, and history.
**Present State Examination**	PSE	Standardized interview for assessing a patient's current mental state.
**Comprehensive Assessment of At-Risk Mental States**	CAARMS	Specifically assesses "prodromal" or at-risk mental states.
**Exner Comprehensive System**	Exner	The standardized system for scoring and interpreting the Rorschach test.
**Signs and Symptoms of Psychotic Illness**	SSPI	Assesses 5 different symptom clusters of psychosis.
**Hamilton Anxiety Rating Scale**	HAM-A	Measures the severity of anxiety symptoms.
**Schedule for Affective Disorders and Schizophrenia**	SADS	Comprehensive diagnostic interview for mood and psychotic disorders.
**Diagnostic Interview Schedule**	DIS	A structured interview used to generate DSM or ICD diagnoses.
**Structured Interview for Schizotypy**	SIS	Assessment of schizotypal personality features and subclinical psychosis.

**LDPS - Child and Adolescent**	LDPS-CA	Child and adolescent version of the Lifetime Dimensions of Psychosis Scale.
**Clinician-Rated Dimensions of Psychosis Symptom Severity**	CRDPSS	Dimensional assessment of psychosis symptoms (DSM-5 tool).
**Krawiecka Scale (Manchester Scale)**	KGV	Assessment of chronic psychotic symptoms, including affective, positive, and negative symptoms.
**Bonn's Scale for Assessment of Basic Symptoms**	BSABS	Assessment of Subtle subjective basic symptoms; used mostly during the prodromal stage of schizophrenia.

Supplementary Table S2: Included items from general psychopathology rating scales

**Scale-Item number**	**Included items**
**BPRS-3**	Depression
**BPRS-14**	Disorientation
**BPRS-15**	Conceptual disorganisation
**BPRS-16**	Blunted affect
**BPRS-18**	Motor retardation
**BPRS-21**	Excitement
**BPRS-22**	Distractibility
**BPRS-23**	Motor hyperactivity
**PANSS-G10**	Disorientation
**PANSS-G11**	Poor attention
**PANSS-G15**	Preoccupation
**PANSS-G7**	Motor retardation
**PANSS-N5**	Difficulty in abstract thinking
**PANSS-N6**	lack of spontaneity and flow of conversation
**PANSS-N7**	Stereotyped thinking
**PANSS-P2**	Conceptual Disorganization
**PANSS-P4**	Excitement
**SANS-9**	Poverty of speech

**SANS-10**	Poverty of speech content
**SANS-11**	Blocking
**SANS-12**	Increased response latency
**SANS-23**	Social Inattentiveness
**SAPS-26**	Derailment
**SAPS-27**	Tangentiality
**SAPS-28**	Incoherence
**SAPS-29**	Illogicality
**SAPS-30**	Circumstantiality
**SAPS-31**	Pressure of speech
**SAPS-32**	Distractible Speech
**SAPS-33**	Clanging

BPRS: BPRS Expanded version; PANSS: Positive and negative syndrome scale; SANS: Scale for assessment of negative symptoms; SAPS: Scale for assessment of positive symptoms.

Supplementary figure S2: Sunburst Map showing the classification of the FTD phenomena.

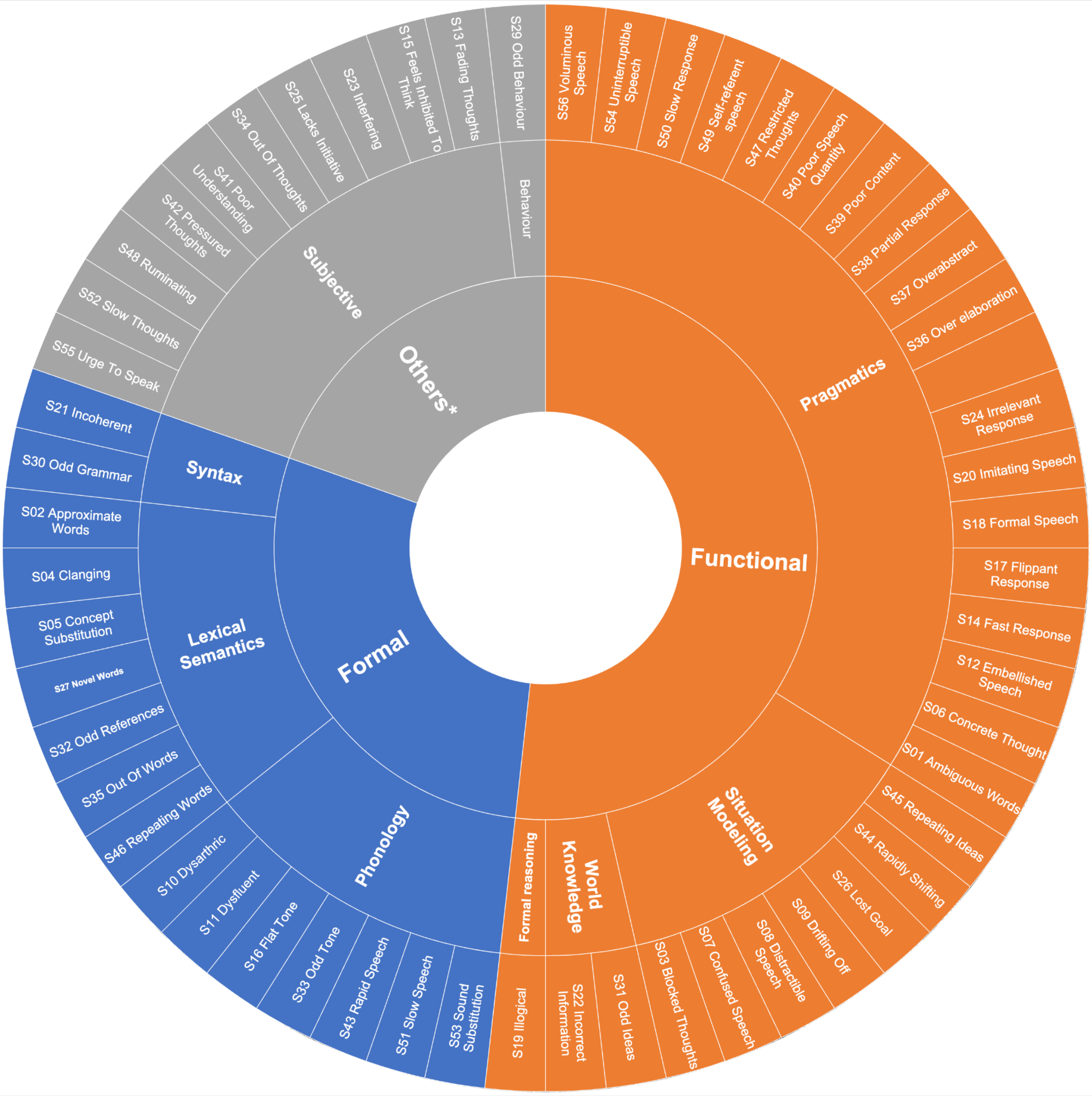

This figure represents the 2 linguistic domains (Functional vs Formal) in the inner layer and their 7 subdomains (Pragmatics, Situation Modelling, World Knowledge, Formal Reasoning, Phonology, Lexicon-semantics, Syntax) in the middle layer. The outer layer enlists all 56 FTD phenomena. *Note that the Subjective FTD Phenomena and Odd behaviour lack a linguistic domain description and are shaded grey.

Supplementary figure S3: Heat maps of coverage of different domains of FTD phenomena by each FTD scale.

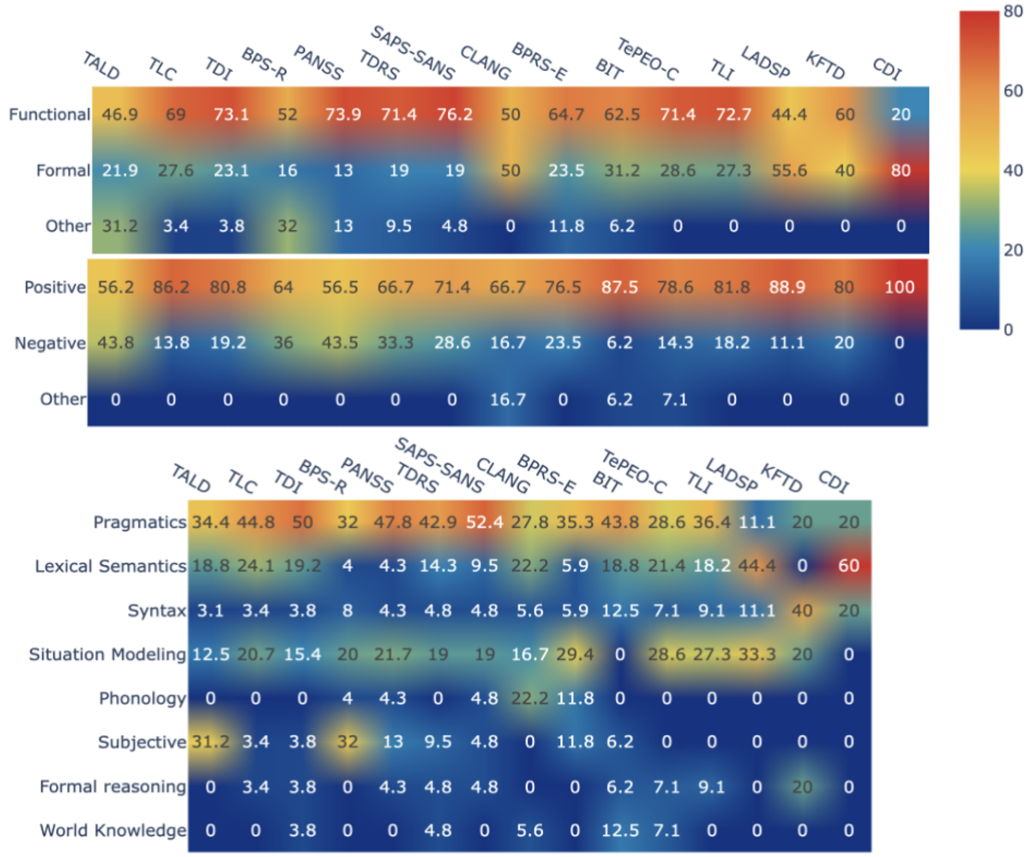

Supplementary Figure S4

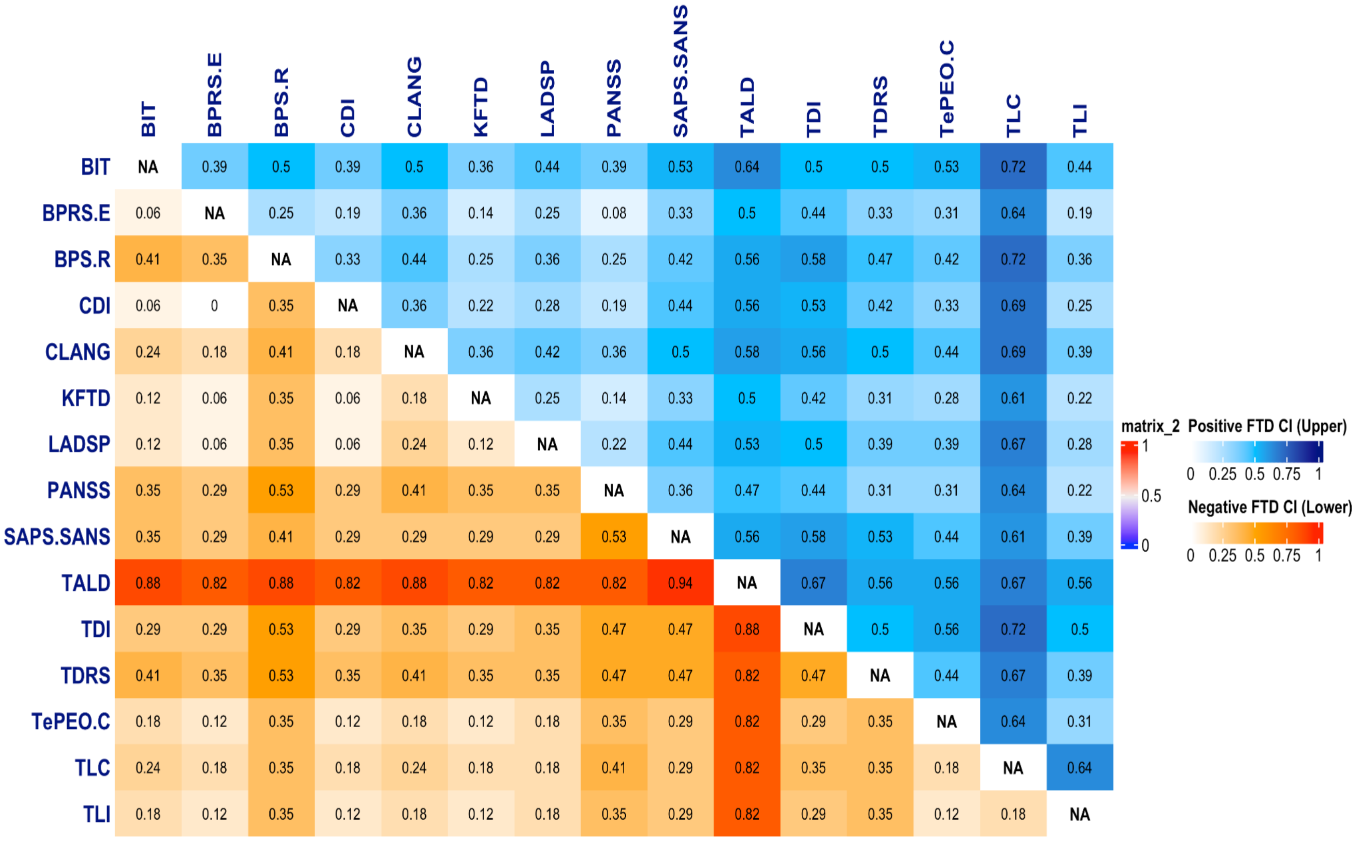

Coverage index for Positive FTD phenomena (Top Right Triangle) and negative FTD Phenomena (Bottom Left Triangle). Combining scales with TLC improves the coverage of positive phenomena and combining scales with TALD seems to improve the coverage of negative phenomena.

Supplementary figure S5: Idiographic radar charts of FTD profiles based on speech transcripts

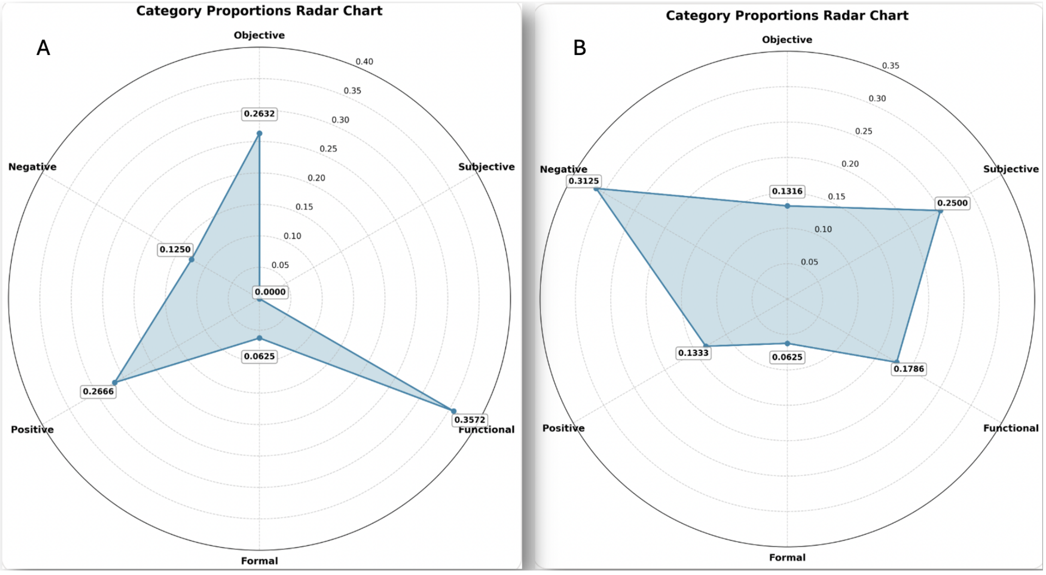

Charts generated using hypothetical transcripts of picture description from two individuals. By providing the information in the Appendix (item list, with definitions and examples) to DeepSeek-R1 (Guo et al., 2025), two picture description transcripts based on TLI short form were 'LLM-rated' for the presence or absence of the 56 FTD phenomena for demonstration purposes. On each domain, the rating was converted into a proportion relative to the number of phenomena out of 56 in Table 1. A. This subject has more positive FTD, with features that are not objective but predominantly affect functional linguistic competence. B. This subject shows high negative FTD and prominent subjective symptoms but has limited positive FTD not much objective features. Images were generated using *matplotlib.pyplot* through scispace.ai.

Charting FTD profiles in this manner also helps to track change in outcome over time or after interventions, for example, by estimating the area within the chart. This also helps to chart the course of the changing profile of FTD across the different phases of illness in patients.

Supplementary Table S3: Top combinations of scales that complement each other to achieve the best coverage of the FTD phenomenon

Scale - Pairs	Coverage		Number of Scale Combinations (Scales)	Coverage
BIT & TALD	70%		6 scales (BIT, BPS-R, CLANG, TALD, TDI, TLC)	98%
CLANG & TALD	70%		6 scales (BIT, BPS-R, CLANG, SAPS-SANS, TALD, TDI)	96%
TALD & TDI	70%		6 scales (BPS-R, CDI, CLANG, TALD, TDI, TLC)	96%
TALD & TLC	68%		5 scales (BPS-R, CLANG, TALD, TDI, TLC)	95%
SAPS.SANS & TALD	64%		4 scales (CLANG, TALD, TDI, TLC)	89%
BPS.R & TALD	63%		4 scales (CLANG, SAPS-SANS, TALD, TDI)	89%
CDI & TALD	61%		4 scales (BPS-R, CLANG, TALD, TDI)	89%
TALD & TDRS	61%		3 scales (CLANG, TALD, TDI)	82%
TALD & TePEO.C	61%		2 scales (3 pairs as in first 2 columns)	70%
TALD & TLI	61%			

Supplementary Material SM1: Mapping Linguistic Domains with FTD

Our observed distribution of greater pragmatic but less formal linguistic competence in FTD scales partially reflects clinical phenomenology: the most striking manifestations of FTD often involve pragmatic breakdown (tangentiality, loss of goal, circumstantiality) and failures in situation modelling (distractibility, derailment) rather than phonological or syntactic errors per se. However, several considerations suggest current coverage may be incomplete: First, while patients with FTD may seem to have intact formal language abilities, subtle abnormalities in lexical selection (e..g, pronoun use; Elleuch, Luo, Chen, & Palaniyappan, 2025a), semantic networks, and syntactic complexity (Elleuch, Luo, Chen, & Palaniyappan, 2025b) have been found through computational linguistic analysis. These features may not be apparent in clinical ratings but could inform mechanisms and outcomes. Furthermore, the functional-formal distinction may be problematic in psychosis. Recent evidence suggests that in schizophrenia, it is difficult to reliably identify patients with purely formal linguistic impairment without functional or pragmatic disturbances, as psychosis inherently involves disordered thinking across both domains. Language and thought are fundamentally intertwined in this context. Third, subjective phenomena (captured by only 10 of 56 items in our analysis, 17.9%) remain underrepresented despite patients' frequent reports of thought pressure, blocking, or interference. Incorporating patient-reported cognitive experiences could provide complementary information to observer-rated speech disturbances.

A more organized conceptualization of FTD measurement might systematically assess: (1) formal linguistic competence (phonology, lexical-semantics, syntax), (2) functional linguistic competence (pragmatics, situation modelling, world knowledge, logical reasoning), and (3) subjective cognitive experiences. Current scales show uneven coverage across these domains, and this is reflected in the 56 items list derived from them. A detailed discussion on FTD can be seen at Palaniyappan, Sreeraj, Venkatasubramanian, & Voppel (2025).

- Elleuch D, Chen Y, Luo Q, Palaniyappan L. Relationship between grammar and schizophrenia: a systematic review and meta-analysis. *Commun Med (Lond)*. 2025;5(1):235. Published 2025 Jun 16. doi:10.1038/s43856-025-00944-1
- Elleuch D, Chen Y, Luo Q, Palaniyappan L. Speaking of yourself: A meta-analysis of 80 years of research on pronoun use in schizophrenia. *Schizophr Res*. 2025;279:22-30. doi:10.1016/j.schres.2025.03.025
- Palaniyappan, L., Sreeraj, V. S., Venkatasubramanian, G., & Voppel, A. (2026). Why is it hard to assess thought disorder? Clarifying the third domain of psychosis. *Schizophrenia Research*, *292*, 41-52.

Supplementary Material SM2

Clinical Assessment of Thought Language And Speech [ATLAS Checklist]

A comprehensive checklist based on content analysis of FTD scales for use during a psychiatric interview to assess 56 distinct FTD phenomena

Assessment Phases
**Phase 1: Initial Observation (Non-verbal)**
☐ Note any unusual behaviors *(#29 Odd Behaviour)*
**Phase 2: Early Interaction (Manner & Attitude)**
☐ Style: Formal? *(#18 Formal speech)*
☐ Style: Flippant/joking? *(#17 Flippant response)*
☐ Style: Self-referent? *(#49 Self-referent speech)*
**Phase 3: Speech Prosody & Rate**
☐ Rate: Fast? *(#43 Rapid Speech)*
☐ Rate: Slow? *(#51 Slow Speech)*
☐ Response latency: Quick? *(#14 Fast Response)*
☐ Response latency: Delayed? *(#50 Slow Response)*
☐ Fluency: Stuttering? False starts? *(#11 Dysfluent)*
☐ Articulation: Poor/slurred? *(#10 Dysarthric)*
☐ Tone: Bizarre quality (pitch/mechanical/volume)? *(#33 Odd Tone)*
☐ Tone: Flat/monotonous? *(#16 Flat Tone)*
☐ Can you interrupt speech? *(#54 if NO)*
**Phase 4: Speech Quantity**
☐ Amount: Too little? *(#40 Poor Speech Quantity)*
☐ Amount: Too much? *(#56 Voluminous Speech)*
☐ Observable thought interruption? *(#3 Blocking)*
**Phase 5: Response Relevance to Question**
☐ Completely unrelated? *(#24 Irrelevant)*
☐ Vaguely related but not direct? *(#28 Oblique)*
☐ Partially addresses the question? *(#38 Partial)*
☐ Never returns to the topic? *(#26 Lost Goal)*

☐ Uncertain about time/place/person? *(#7 Confused Speech)*
**Phase 6: Quality of Expressed Ideas**
☐ Sufficient words but lacking ideas? *(#39 Poor Content)*
☐ Topics restricted to a few themes? *(#47 Restricted Thoughts)*
☐ Excessively lively/over-described? *(#12 Embellished)*
☐ Tedious, long-winded, delayed point? *(#36 Overelaboration)*
☐ Provides false/incorrect information? *(#22 Incorrect Info)*
☐ Ideas defy social conventions? *(#31 Odd Ideas)*
**Phase 7: Flow & Organization**
☐ Ideas shift rapidly with weak connections? *(#44 Rapidly Shifting)*
☐ Ideas gradually drift from the start? *(#9 Drifting Off)*
☐ Same themes keep recurring? *(#45 Repeating Ideas)*
☐ Distracted by external stimuli? *(#8 Distractible)*
☐ Echoes the interviewer's words? *(#20 Imitating)*
☐ Same words repeated? *(#46 Repeating Words)*
**Phase 8: Sentence Structure & Grammar**
☐ Words arranged fail to convey meaning? *(#21 Incoherent)*
☐ Sentence construction is abnormal, but understandable? *(#30 Odd Grammar)*
☐ Pronouns/articles have unclear referents? *(#32 Odd References)*
☐ Conclusions don't follow from premises? *(#19 Illogical)*
**Phase 9: Word Choice & Semantics**
☐ Completely invented new words, approximate words or unusual use of known words? *(#1, 2 or 27 Ambiguous, Approximate or Novel Words)*
☐ Related concept substituted? *(#5 Concept Substitution)*
☐ Ideas associated by sound (rhymes/puns)? *(#4 Clanging)*
☐ Difficulty finding correct word? *(#35 Out Of Words)*
☐ Mispronunciation of words? *(#53 Sound Substitution)*
**Phase 10: Abstract Thinking (Test with proverbs/categories)**
☐ Can only interpret literally? *(#6 Concrete Thought)*
☐ Too general, overinclusive without boundaries? *(#37 Overabstract)*

Phase 11: Subjective Experiences (Requires Direct Inquiry)
**A. Thought Generation Problems**
☐ Patient reports: "My thoughts are disappearing" *(#13 Fading)*
☐ Patient reports: "I have no thoughts/ideas" *(#34 Out Of Thoughts)*
☐ Patient reports: "It's hard to think/resistance" *(#15 Feels Inhibited)*
☐ Patient reports: "Can't start thinking" *(#25 Lacks Initiative)*
☐ Patient reports: "My thoughts are very slow" *(#52 Slow Thoughts)*
**B. Excessive/Intrusive Thoughts**
☐ Patient reports: "Too many thoughts, can't control" *(#42 Pressured)*
☐ Patient reports: "Strange thoughts keep intruding" *(#23 Interfering)*
☐ Patient reports: "Strong urge to keep talking" *(#55 Urge To Speak)*
☐ Patient reports: "Can't stop thinking about [unpleasant]" *(#48 Ruminating)*
**C. Subjective Comprehension Difficulties**
☐ Patient reports: "I don't understand what words mean" *(#41 Poor Understanding)*

Notes:

- Phases 1-10 are based on observable features
- Phase 11 requires direct patient self-report
- Phenomena #10, #11, #16, #33, #43, #51 are best assessed with audio recording
- This checklist identifies the presence of phenomena, not its severity
